# Antimicrobial Resistance Trends Among Obligate Anaerobic Bacteria Isolated From Chronic Suppurative Otitis Media Patients in a Tertiary Care Teaching Hospital in Central India

**DOI:** 10.7759/cureus.92221

**Published:** 2025-09-13

**Authors:** Arati A Bhadade, Farha Siddiqui, Utkal Mishra, Shaila Sidam, Ritika Chouhan, Richa Tiwari, Vaishnavi Pichaikran, Shashwati Nema

**Affiliations:** 1 Microbiology, All India Institute of Medical Sciences, Bhopal, Bhopal, IND; 2 Otorhinolaryngology (ENT-Head and Neck Surgery), All India Institute of Medical Sciences, Bhopal, Bhopal, IND; 3 Biotechnology, Career College, Bhopal, Bhopal, IND

**Keywords:** anaerobes, antimicrobial resistance, antimicrobial susceptibility testing, chronic suppurative otitis media, obligate anaerobic bacteria

## Abstract

Introduction: Chronic suppurative otitis media (CSOM) remains a major public health concern, especially in low- and middle-income countries, contributing significantly to preventable hearing loss. While aerobic bacteria are well-recognized pathogens in CSOM, anaerobic bacteria’s role is increasingly acknowledged due to improved sampling and culture techniques. Anaerobes may synergize with aerobes, worsening disease severity and complicating management. This study aimed to identify the anaerobic bacterial profile in CSOM patients and assess antimicrobial susceptibility patterns of these isolates.

Methods: A 12-month observational cross-sectional study was conducted at a tertiary care center in Central India, enrolling 100 patients aged >12 years with clinically suspected CSOM and chronic purulent ear discharge lasting ≥6 weeks. Pus samples were collected aseptically, inoculated in Robertson’s cooked meat medium, and processed for anaerobic culture following standard protocols. Isolates were identified using matrix-assisted laser desorption/ionization-time of flight (MALDI-TOF; VITEK® MS PRIME, bioMérieux, France). Antimicrobial susceptibility testing (AST) of obligate anaerobes was performed by the E-test for metronidazole, clindamycin, ciprofloxacin, amoxicillin-clavulanic acid, and imipenem.

Results: Obligate anaerobes were isolated in 12% of cases, with Gram-positive anaerobes predominating. *Finegoldia magna* and *Bacteroides fragilis* were the most common species. The AST pattern varied between Gram-positive and Gram-negative obligate anaerobes. Clindamycin showed the highest susceptibility (84.6%), whereas ciprofloxacin demonstrated the lowest (38.5%).

Conclusion: Anaerobic culture and susceptibility testing are critical in managing CSOM, especially in refractory cases. Empirical use of ciprofloxacin should be cautious due to limited anaerobic coverage. Clindamycin and amoxicillin-clavulanic acid offer more reliable treatment options, potentially improving outcomes and mitigating antimicrobial resistance.

## Introduction

Chronic suppurative otitis media (CSOM) is defined as a chronic inflammation of the middle ear and mastoid mucosa, accompanied by otorrhea (ear discharge) persisting for at least six weeks [1]. Affected patients often present with a perforated tympanic membrane, which may occur spontaneously due to the underlying pathology [2]. CSOM remains a significant public health issue, particularly in low- and middle-income countries, where it contributes substantially to preventable hearing loss [3]. Because of the chronic nature of CSOM, determining its exact prevalence is challenging. Nonetheless, the global burden is substantial, affecting an estimated 65-330 million individuals, primarily in developing regions. According to the World Health Organization (WHO), the prevalence of CSOM in India is approximately 7.8%, classifying it among countries with a very high prevalence [4]. A recent meta-analysis by Onifade et al. reported CSOM global prevalence of 3.8% of the global population, 85% of whom are from low- and middle-income countries [5]. Bhatia et al. reported in a meta-analysis a 3.78% pooled estimated prevalence of CSOM in children in India [6].

CSOM is broadly classified into mucosal and squamous types, with varying degrees of severity and complications depending on the subtype [7]. Potential complications include conductive hearing loss, typically due to ossicular chain disruption [2]; vertigo, from inner ear involvement [7]; persistent headache, potentially indicating intracranial involvement [7]; extracranial complications, such as subperiosteal abscess, labyrinthitis, mastoiditis, and facial paralysis [2,7]; and intracranial complications, including meningitis, cerebral abscess, encephalitis, extradural abscess, lateral sinus thrombosis, and otic hydrocephalus [2,7]. These life-threatening complications warrant timely diagnosis and appropriate management of CSOM.

Understanding the microbiological profile of CSOM cases is crucial for accurate diagnosis and effective treatment. CSOM is often polymicrobial in nature, involving both aerobic and anaerobic bacteria and occasionally fungi [8]. While aerobic bacteria are well-documented in CSOM, the role of anaerobic organisms has gained increasing attention with advancements in sampling techniques, transport methods, and culture practices [8-10]. Chronic, non-resolving CSOM with a foul-smelling purulent discharge should raise clinical suspicion for anaerobic infection [10]. Anaerobic bacteria, which are part of the normal flora of the oropharyngeal mucosa, can reach the middle ear via the eustachian tube under pathological conditions [9]. These anaerobes may act synergistically with aerobic pathogens, exacerbating disease severity.

Effective treatment requires culture-directed therapy and identification of antibiotic resistance patterns. The rise of multidrug-resistant (MDR) organisms, including anaerobes, is a growing concern [11]. Notably, many anaerobes, including members of the *Bacteroides fragilis *group, produce β-lactamases, leading to treatment failures [10]. Increasing resistance among anaerobic Gram-negative bacilli such as *Prevotella, Porphyromonas, and Fusobacterium *spp. has also been observed [12].

Despite extensive research on aerobic organisms in CSOM, limited studies have investigated the role of anaerobic bacteria. Given their significant contribution to pathogenesis and potential for resistance, this study aims to identify the anaerobic bacterial profile among CSOM patients and assess the antimicrobial susceptibility patterns of these anaerobic isolates. This approach will aid in optimizing treatment regimens and mitigating antibiotic resistance, thereby reducing morbidity associated with CSOM.

## Materials and methods

Design and setting

A prospective cross-sectional study was conducted over 12 months from June 2024 to May 2025 in the Departments of Microbiology and ENT at a tertiary care institute in Madhya Pradesh, India. Ethical approval was obtained from the Ethical Review Board of our institute (reference number: ID-IHEC-LOP/2024/P24/039).

Study population

Patients presenting to the ENT outpatient department (OPD) with the chief complaint of chronic purulent ear discharge for a duration of ≥ 6 weeks (chronic suppurative otitis media) and above 12 years of age were enrolled for the study. Patients with previous ear surgeries and on topical antibiotics were excluded from the study. Written informed consent was obtained from all the recruited study participants.

Sample size

The calculation of sample size was 111, taking a 7.8% prevalence for anaerobic bacteria, 5% marginal error, and a 95% confidence interval. We got polymicrobial flora in 11 samples; hence, we did not include them in this study.

Procedure

The external auditory canal was cleaned with povidone-iodine, followed by 70% alcohol using sterile cotton swabs. The canal was allowed to dry for three minutes after alcohol application [9,13]. Middle ear exudate was aspirated using a syringe needle inserted through the tympanic membrane perforation under light source guidance. Care was taken to avoid contamination from the external auditory canal. A portion of the aspirate was immediately inoculated into Robertson’s cooked meat medium (RCM). When the exudate volume was insufficient, swabs with anaerobic transport medium were used for sample collection. The samples were transported immediately to the microbiology laboratory for processing.

For anaerobic culture and identification, subcultures from enriched RCM were plated onto Brucella blood agar (BBA) and neomycin blood agar (NBA) with a 5 µg metronidazole disc and incubated anaerobically for 48-72 hours [14]. An automated anaerobic gas filling system, Anoxomat (Don Whitley Scientific, Bingley, England), was used for anaerobic culture processing. The isolates were presumptively identified by Gram staining and aerotolerance testing. Final identification was confirmed using MALDI-TOF MS (VITEK® MS PRIME, bioMérieux, France) for obligate and facultative anaerobes. Antimicrobial susceptibility of confirmed obligate anaerobic isolates was performed by the Epsilometer test (E-test) method for metronidazole, clindamycin, ciprofloxacin, amoxicillin-clavulanic acid, and imipenem. Results were interpreted based on the Clinical and Laboratory Standards Institute (CLSI) M100-S34 criteria [15,16].

Data collection and statistical analysis

Statistical analysis was done by using the chi-square test. Quantitative variables, such as age, were expressed as mean ± standard deviation. Qualitative variables, including gender, bacterial isolates, and antimicrobial susceptibility patterns, were reported as frequencies and percentages. The p-value of ≤ 0.05 was considered significant.

## Results

A total of 100 patients diagnosed with CSOM were recruited during the study period. Twenty-one samples were swabbed due to insufficient exudate. The majority (80%) were adults, with no significant gender predilection. Notably, 20% of cases were observed in the 12-18-year-old age group. Hearing loss was the most frequently reported symptom, present in 51% of patients, followed by ear pain in 39%. Common risk factors identified in the study included immunocompromised conditions, such as malnutrition, steroid use, and diabetes mellitus (Table 1).

**Table 1:** Clinical and demographic characteristics of the recruited study participants (n = 100) CSOM: Chronic suppurative otitis media

Clinico-Demographic Details	Parameter	CSOM patients with an obligate anaerobic infection (n = 12, 11 monomicrobial and 1 polymicrobial)	CSOM patients without an obligate anaerobic infection (n = 88)	p-value	
Age Distribution	< 18 yrs (20)	3 (15%)	17 (85%)	0.64	
	>18 yrs (80)	9 (11.25%)	71 (88.75%)		
Gender Distribution	Female	6 (12.5%)	42 (87.5%)	0.88	
	Male	6 (11.53%)	46 (88.46%)		
Clinical Features	Hearing loss	8 (15.68%)	43 (84.31%)	0.38	
	Ear pain	6 (15.38%)	33 (84.61%)	0.88	
	Vertigo	5 (25%)	15 (75%)	0.03	
	Headache	5 (27.77%)	13 (72.22%)	0.15	
Risk Factors	Malnutrition	3 (17.64%)	14 (82.35%)	0.57	
	Steroid intake	1 (14.28%)	6 (85.71%)	0.38	
	Diabetes mellitus	1 (50%)	1 (50%)	0.24	

**Figure 1:**
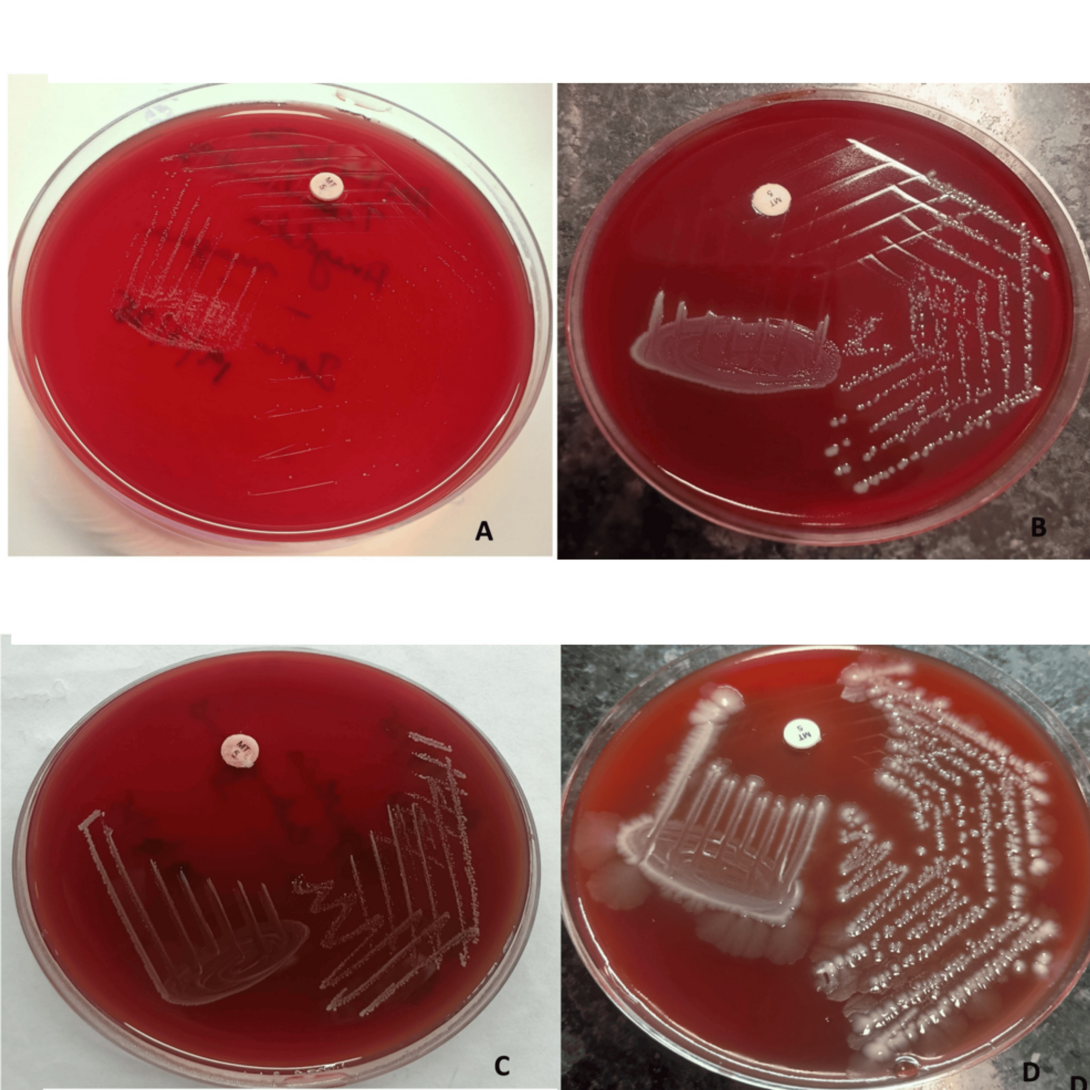
Anaerobic isolates showing growth on neomycin blood agar (NBA) (a) *Finegoldia magna*, b) *Bacteroides fragilis*, c) *Eggerthia catenaformis*, d) *Clostridium perfringens*

Obligate anaerobes were isolated in 13 out of 100 (13%) recruited CSOM cases (12 cases, 11 monomicrobial, and one polymicrobial), either as monomicrobial or polymicrobial growth. Gram-positive obligate anaerobes were the most commonly isolated group. The most frequently identified anaerobic organism was *Finegoldia magna *(n = 5), followed by *B. fragilis* (n = 3). Other anaerobic isolates included *Clostridium perfringens, Clostridium sporogenes, Clostridium novyi, Eggerthia catenaformis, and Prevotella bivia* (Table 2 and Figure 1).

**Table 2:** Distribution of anaerobic isolates

Sr. No	Growth type	Name of Isolated Organisms	
Polymicrobial		Isolates	N (%)
	1. Obligate and facultative anaerobes	*Finegoldia magna *and *Proteus mirabilis*	1 (7.7%)
		*Finegoldia magna* and *Staphylococcus aureus*	1 (7.7%)
		*Bacteroides fragilis* and *Escherichia coli*	1 (7.7%)
		*Bacteroides fragilis* and *Morganella morganii*	1 (7.7%)
		*Clostridium perfringens* and *Enterococcus faecium*	1 (7.7%)
	2. Obligate anaerobes	*Finegoldia magna* and *Eggerthia catenaformis*	2 (15.3%)
Total obligate anaerobes		7	53.80%
Monomicrobial		Isolates	N (%)
	Obligate anaerobes	Finegoldia magna	2 (15.3%)
		Clostridium sporogenens	1 (7.7%)
		Clostridium novyi	1 (7.7%)
		Bacteroides fragilis	1 (7.7%)
		Prevotella bivia	1 (7.7%)
Total obligate anaerobes		6	46.10%

**Figure 2:**
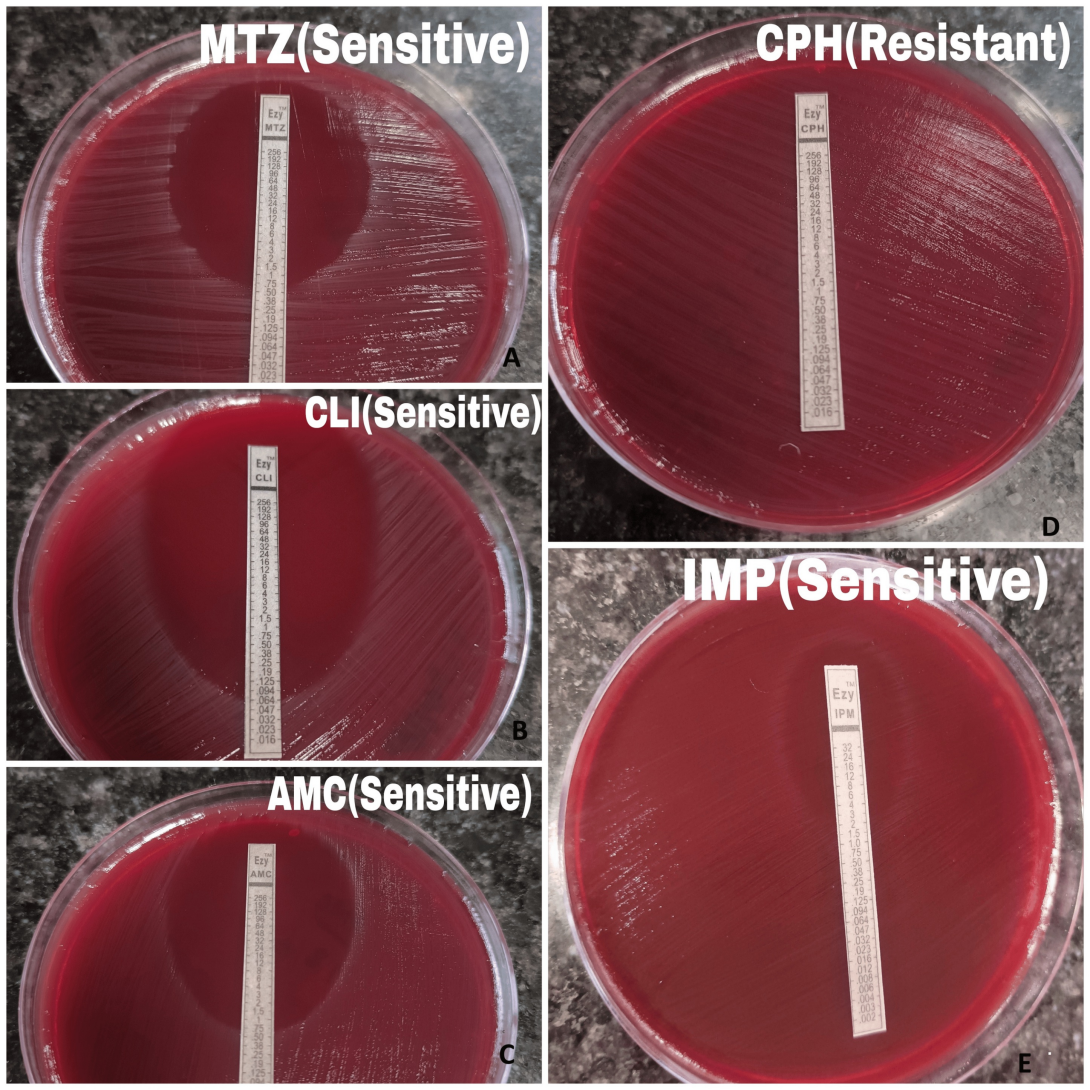
Antimicrobial susceptibility testing (AST) pattern of obligate anaerobic isolates by the E-strip method Tested for A) metronidazole (MTZ), B) clindamycin (CLI), C) amoxicillin-clavulanic acid (AMC), D) ciprofloxacin (CPH), and E) imipenem (IMP)

The AST pattern varied between Gram-positive and Gram-negative obligate anaerobes. Gram-positive anaerobic isolates demonstrated the highest susceptibility to metronidazole, amoxicillin-clavulanic acid, and imipenem while showing the least susceptibility to ciprofloxacin. Gram-negative obligate anaerobes exhibited good susceptibility to clindamycin, but a high resistance rate was observed for metronidazole, imipenem, and ciprofloxacin. Overall, the highest susceptibility among all anaerobic isolates was noted for clindamycin (84.6%), while the lowest was for ciprofloxacin (38.5%) (Table 3 and Figure 2).

**Table 3:** Antimicrobial susceptibility testing (AST) pattern of obligate anaerobic isolates by the E-strip method S = Sensitive, R = Resistant No isolate was found intermediate resistant.

Organisms	Clindamycin		Metronidazole		Amoxycillin-Clavulanic Acid		Imipenem		Ciprofloxacin	
	S	R	S	R	S	R	S	R	S	R
Gram-positive anaerobes										
*Finegoldia magna* (n=5)	3	2	4	1	5	0	5	0	1	4
*Clostridium sporogenes* (n=1)	1	0	1	0	1	0	1	0	1	0
*Clostridium perfringens* (n=1)	1	0	1	0	1	0	1	0	1	0
*Clostridium novyi* (n=1)	1	0	1	0	0	1	0	1	0	1
*Eggerthia catenaformis* (n=1)	1	0	1	0	1	0	1	0	1	0
Total (n=9) (69.24%)	7 (77.8%)	2 (22.2%)	8 (88.9%)	1 (11.1%)	8 (88.9%)	1 (11.1%)	8 (88.9%)	1 (11.1%)	4 (44.5%)	5 (55.5%)
Gram-negative anaerobes										
*Bacteroides fragilis* (n=3)	3	0	0	3	2	1	1	2	1	2
*Prevotella bivia* (n=1)	1	0	1	0	0	1	0	1	0	1
Total (n=4) (30.76%)	4 (100%)	0 (0%)	1 (25%)	3 (75%)	2 (50%)	2 (50%)	1 (25%)	3 (75%)	1 (25%)	3 (75%)
Grand Total (n=13)	11	2	9	4	10	3	9	4	5	8
Percentage (%)	84.6%	15.4%	69.3%	30.7%	76.9%	23.1%	69.3%	30.7%	38.5%	62.5%

## Discussion

CSOM is a major contributor to hearing loss and health complications globally, particularly in developing nations. Although aerobic bacteria have traditionally been recognized as the primary pathogens in CSOM, the importance of anaerobic bacteria has become increasingly evident in recent years. Gaining insight into the prevalence, types, and antibiotic sensitivity of these anaerobic bacteria is essential for optimizing treatment strategies and ensuring targeted antibiotic therapy.

The present study found a higher frequency of CSOM among adult patients. This aligns with the findings of Chirwa et al. [17], who reported a peak incidence of 61.5% in individuals over 18 years old, and Hiremath et al. [18], who observed a 51% peak incidence in the 21-60 years age group. CSOM is commonly associated with symptoms such as hearing loss, ear pain, vertigo, and headache. In our study, hearing loss was the most prevalent symptom, occurring in 51% of patients, followed by ear pain (39%) and vertigo (20%). These results are consistent with those reported by Bhat et al. [19]. No significant differences in clinical presentations were noted between CSOM patients with and without anaerobic infections, except for vertigo. This can be attributed to the chronic, often insidious nature of anaerobic infections, which can cause persistent damage and a prolonged inflammatory response, extending beyond the middle ear to involve the inner ear. The low-oxygen environment created by anaerobes may also promote the formation of biofilms, making the infection more resistant to treatment and allowing ongoing irritation of the inner ear, which can contribute to vertigo. Common risk factors identified included immunocompromised states, such as malnutrition, steroid use, and diabetes mellitus. Supporting this, Adebola et al. reported that patients with compromised immunity had a significantly higher rate of anaerobic bacterial isolation from chronic middle ear discharge [9] (Table 1).

In our study, we observed an equal distribution between monomicrobial infections, involving a single obligate anaerobe, and polymicrobial infections, where obligate anaerobes coexisted with facultative anaerobes. Notably, one case exhibited a polymicrobial infection with two anaerobic species, *F. magna* and *E. catenaformis* (Table 2). These findings are consistent with previous studies by Srivastava et al. [20] and Prakash et al. [21]. Polymicrobial infections involving anaerobic bacteria are common in CSOM, as the chronic nature of the infection creates an anaerobic environment conducive to the growth of anaerobic pathogens. There is substantial evidence supporting a synergistic relationship between aerobic and anaerobic bacteria in chronic infections, which often renders polymicrobial infections more virulent and difficult to treat than monomicrobial infections [22]. This synergy may enhance bacterial survival, increase resistance to host defences, and complicate clinical outcomes. Further elaboration on these mechanisms will deepen understanding of the pathogenicity of polymicrobial infections in CSOM.

The present study identified a 13% prevalence of anaerobic bacteria in CSOM patients (Table 2), which closely corresponds with the 10.2% reported by Adebola et al. [9]. However, isolation rates reported in earlier studies have varied significantly, ranging from as low as 3.5% [20] to as high as 33.6% [17]. This variability may be due to differences in sample collection methods, the handling and processing of specimens under strict anaerobic conditions, the anatomical site and technique used for sample acquisition (e.g., tubotympanic versus atticoantral), regional differences in microbial flora, and patients’ prior antibiotic exposure.

Gram-positive obligate anaerobes were the most frequently isolated group among CSOM patients in our study (Table 3). *F. magna *emerged as the predominant anaerobic species, followed by *Clostridium *species. Neeff et al. investigated the microbial composition and absolute abundance of clinically relevant bacteria in tissue samples from CSOM patients with cholesteatoma, finding a predominance of *Anaerococcus *and *Peptoniphilus *species [23]. Additionally, Gram-negative obligate anaerobes, such as *B. fragilis *and *P. bivia*,* *were isolated in our study (Table 3), consistent with the observations reported by Chirwa et al. [17] and Geeta et al. [24].

In the present study, antimicrobial susceptibility patterns varied between Gram-positive and Gram-negative obligate anaerobes. Gram-positive anaerobes showed good susceptibility to metronidazole, amoxicillin-clavulanic acid, and imipenem. In contrast, Gram-negative obligate anaerobes were generally susceptible to clindamycin but exhibited high resistance rates to metronidazole and imipenem. Overall, clindamycin demonstrated the highest efficacy against all anaerobic isolates, while ciprofloxacin showed the lowest susceptibility (Table 3). Similar findings were reported by Adebola et al., who observed 100% metronidazole sensitivity among Gram-negative anaerobes but only 62.5% sensitivity in Gram-positive anaerobes tested [9]. Although metronidazole and ciprofloxacin are commonly used as empirical treatments for CSOM, our study revealed significant resistance to these agents (Table 3). These results highlight the critical need for isolating anaerobic bacteria and performing antimicrobial susceptibility testing to enable clinicians to tailor antibiotic therapy effectively for each patient. The main limitation of this study is that it was a single-center pilot study with a small sample size. It may not fully represent the regional variability in antimicrobial resistance patterns. Additionally, convenience sampling and challenges in optimal anaerobic sample collection could have influenced the spectrum and susceptibility profile of isolates, potentially limiting the generalizability and robustness of our findings. We emphasize the need for future research through multicentric studies involving a larger and more representative population to validate and expand upon these findings.

## Conclusions

This study indicated a 13% prevalence of obligate anaerobic bacteria in CSOM patients, underscoring a significant burden of anaerobic participation in these diseases. CSOM patients with anaerobic infections had vertigo much more frequently, which may be a clinical marker of the severity of anaerobic infections. Reducing morbidity, enhancing clinical outcomes, and halting the emergence of resistance all depend on precise anaerobic pathogen identification and focused antibiotic treatment. Despite being a widely used empirical agent, ciprofloxacin should be used with caution due to its low effectiveness against anaerobes. When anaerobic infections are detected, clindamycin and amoxicillin-clavulanic acid are advised because they offer more dependable coverage. These results highlight the significance of using susceptibility testing and anaerobic culture in the diagnostic workup of CSOM, particularly in patients who are not responding to empirical treatment.
